# Inside‐Out Surgical Anatomy of Superior Laryngeal Artery Endoscopic Dissection and Proposal for Nomenclature of Branches

**DOI:** 10.1002/oto2.42

**Published:** 2023-04-16

**Authors:** Hazem M.A. Saleh, Thomas Jöns, Dirk Mürbe, Tadeus Nawka

**Affiliations:** ^1^ Otorhinolaryngology Unit, Department of Medical Applications of Laser, National Institute of Laser Enhanced Sciences Cairo University Giza Egypt; ^2^ Klinik für Audiologie und Phoniatrie, Department of Audiology and Phoniatrics Charité—Universitätsmedizin Berlin Berlin Germany; ^3^ Berliner Simulations & Trainingszentrum (BeST), Department of Anatomy Charité—Universitätsmedizin Berlin Berlin Germany

**Keywords:** inside‐out, superior laryngeal artery

## Abstract

**Objectives:**

To describe the inside out surgical anatomy of the superior laryngeal artery and to resolve the ambiguities in the nomenclature of its main branches.

**Study Design:**

Endoscopic dissection of the superior laryngeal artery in the paraglottic space of larynges of fresh frozen cadavers and a review of the literature.

**Setting:**

A center for anatomy encompassing facilities for latex injection into the cervical arteries of human donor bodies and a laryngeal dissection station equipped with a video‐guided endoscope and a 3‐dimensional camera.

**Methods:**

Video‐guided endoscopic dissection of 12 hemilarynges in fresh frozen cadavers whose cervical arteries were injected with red latex. Description of the inside‐out surgical anatomy of the superior laryngeal artery and its main branches. Review of the previous reports describing the anatomy of the superior laryngeal artery.

**Results:**

From inside the larynx, the artery was exposed upon its entry through the thyrohyoid membrane or through the foramen thyroideum. It was traced ventrocaudally in the paraglottic space exposing its branches to the epiglottis, the arytenoid, and the laryngeal muscles and mucosa. Its terminal branch was followed until it left the larynx through the cricothyroid membrane. Branches of the artery, previously described under different names, appeared to supply the same anatomical domains.

**Conclusion:**

Mastering the inside out anatomy of the superior laryngeal artery is mandatory to control any intraoperative or postoperative hemorrhage during transoral laryngeal microsurgery or during transoral robotic surgery. Naming the artery's main branches according to their domain of supply would resolve the ambiguities resulting from various nomenclatures.

The larynx receives its arterial blood supply from 3 different sources: the superior laryngeal artery (SLA), the cricothyroid branch of the superior thyroid artery, and the inferior laryngeal artery (ILA). The SLA arises in most of the cases from the superior thyroid artery and the ILA from the inferior thyroid artery.^1^, ^2^, ^3^ The SLA is a dominant nutrient artery of the larynx.^1^ Only one SLA can provide blood supply to the whole larynx through free anastomoses with its contralateral fellow artery and with the inferior laryngeal arteries on both sides.^4^, ^5^

Pertinent knowledge of the anatomy of the SLA and its main branches from the endolaryngeal vantage point is mandatory to manage any arterial hemorrhage during or after transoral laryngeal microsurgery (TLM) or transoral robotic surgery (TORS). The lack of standardized naming of the intralaryngeal branches of the SLA and the absence of their representation in the Nomina Anatomica^2^ created discrepancies between authors^3^ regarding the anatomical description of the main branches of the SLA.

Oki first described the anatomy of the SLA in 1958. He studied the arterial distribution of 120 hemilarynges by X‐ray stereogram after injecting a radio‐opaque material into the arteries. He described 5 branches of the SLA and named them the ascending branch, the descending branch, the dorsal branch, the ventral branch, and the medial branch. He cited 7 different types of distribution of the SLA in the larynx, 5 patterns of vascularization of the epiglottis, and 4 patterns of vascularization of the arytenoid.^4^

Pearson in 1975, microdissected 20 hemilarynges whose vessels were injected with colloidal radiopaque particles. He adopted the same branches' naming suggested by Oki. He added that the descending branch of the SLA splits into an anterior and a posterior division.^5^ Later, Sato used also the same branches' nomenclature adopted by Oki and Pearson.^6^

Tissues and organs having the capacity to vibrate require adapted vascular structures to overcome the risk of hypoxia from blood flow interruption.^6^ Accordingly, undulation or meandering is a feature of some branches of the SLA.^4^ Imura et al,^7^ assessed the meandering of some branches of the SLA in 26 hemilarynges of female cadavers. They described 6 branches of the SLA and called them the superior posterior, the anterior, the medial posterior, the medial, the antero‐inferior, and the postero‐inferior branches.

Rusu et al,^2^ did intralaryngeal dissection of 32 hemilarynges, after removing the laminae of the thyroid cartilages and separating the cricothyroid joint. They advocated that the SLA exhibits 5 constant branches and termed them the superior, the anterior, the postero‐medial, the antero‐inferior and the postero‐inferior branches.

Imanishi et al,^1^ demonstrated the SLA with angiograms on 3 fresh cadavers injected with a radiopaque material in the femoral and common carotid arteries. They described the SLA as having only an ascending and a descending branch, the descending branch ending into an anterior and a posterior division.

Goyal et al,^3^ used a surgical robot to perform a transoral dissection of the supraglottic region in 5 fresh frozen cadaveric heads vascularly injected with silicone. They adopted the same nomenclature of the branches of the SLA as Rusu et al,^2^ and cited the anterior, the superior, and the posteromedial branches.

Perotti et al performed in 2018 a microdissection of 11 fresh frozen cadavers tracing the course of the laryngeal vessels from outside inwards, after removal of the thyroid cartilage. In their work, they described only 3 branches of the SLA, namely the epiglottic artery, the postero‐inferior artery, and the antero‐inferior artery. They tagged the last 2 branches as the terminal branches of the main trunk of the SLA. According to these authors, the postero‐inferior artery divides in turn into 2 terminal branches. Perotti et al were the first to advocate the presence of 2 arterial anastomotic networks in the larynx, a medial and a lateral one, connecting the antero‐inferior artery to the postero‐inferior artery.^8^

In the actual study, we associated the technique of injection of red latex into the cervical arteries^9^ of the human fresh cadavers to the inside‐out endoscopic dissection of the larynges while in the surgical position. Our first aim was to offer a real description of the inside out surgical anatomy of the SLA and its main branches. Second, we aimed to resolve the ambiguities in the nomenclature of the main branches of the SLA, through reviewing and comparing the previous relevant reports on the surgical anatomy of the SLA and its main branches.

## Methods

The research was exempt from the approval of the institutional review board (IRB) since it was on cadavers from body donors, who have provided their personal consent in a written agreement to the use of their body after their death with the Centre for Anatomy, Charité—Universitätsmedizin Berlin. The exemption was issued by the ethics committee at the Charité—Universitätsmedizin Berlin.

Six fresh cadavers of human body donors were transferred to the Center for Anatomy, Charité—Universitätsmedizin Berlin. Their race, age, sex, and cause of death were noted. The carotid arteries on both sides were meticulously dissected.^10^ Breach of the vessels or their branches were avoided. Plastic tubes/cannulas were introduced into the common carotid artery on each side through a small incision secured by sutures. The internal carotids were ligated at the skull bases. We flushed the arterial system of the necks with saline to remove any debris or clots. Leaking points, if any, were identified and secured by a hemostat. Twenty to 45 cc of red latex (Ward Science) were injected into the arterial system of the necks. The injection continued until the red latex spilled out from the plastic tubes/cannulas of the contralateral sides.

After injection, the cadavers were frozen for at least 48 hours at −20°C. Once the latex had solidified, the necks were transected at the level of C7‐T1 and stored at −20°C until the start of the transoral video‐guided endoscopic dissection of the larynges.

Each specimen was dissected 3 times in average on 3 nonconsecutive days. Before each session, the specimens were allowed to defrost at room temperature for 20 hours. Each dissection session lasted for 5 hours in average. After each session, the heads were refrozen at −20°C for at least 24 hours.

The cadaveric heads were put in a position similar to the surgical position during TLM with the necks hyperextended. The light guide was used to position the laryngoscope. The laryngoscope displaced the epiglottis ventrally and was suspended by a support. We used a special laryngoscope (Spiggle & Theis) equipped with a whole length socket encompassing a 0°, 14 cm long endoscope mounted to an EndoSURGERY 3D Spectar camera (Xion GmbH).

The larynges were dissected from the inside out using microphonosurgical instruments (Spiggle & Theis). The dissection started, under videoendoscopic guidance, by an incision in the mucosa of the aryepiglottic fold. The adipose tissue was extracted from the paraglottic space and the adjacent pre‐epiglottic space by a mixture of sharp and blunt dissection while preserving the muscles and neurovascular structures.

The SLA and its branches were dissected and traced in the paraglottic space from the dorso‐cranial entry point to the ventro‐caudal exit point. Dissections were video‐recorded and photos were shot. Photos and videos were reviewed several times. We reviewed the previous relevant reports describing the anatomy of the SLA and compared the different nomenclatures of the branches of the SLA in different reports.

## Results

The body donors' ages of death ranged from 73 to 98 years (mean = 85 years). They were 4 females and 2 males, all Caucasians. They all died from systemic conditions and were not suffering from any known laryngeal disease.

The red latex filled the superior laryngeal arterial system in 9 out of the 12 hemilarynges. The SLA was seen entering the paraglottic space at the dorso‐cranial part of its lateral wall by piercing the thyrohyoid membrane in 10 hemilarynges and through a foramen thyroideum in 2 hemilarynges, 1 on each side, in 2 different heads. To note that Oki reported the entry of the SLA through a foramen thyroideum in 20% of his specimens.^4^ From the endolaryngeal aspect, we found that the point of entry of the SLA was surrounded in all 12 hemilarynges by a significant amount of fat, more abundant than the cushion of fat surrounding the artery during its entire course.

**Figure 1:**
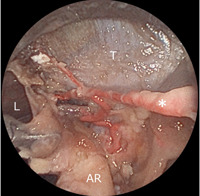
The main trunk of the right SLA (*) descending obliquely in a ventro‐caudal direction. AR, right arytenoid area; L, laryngeal lumen; SLA, superior laryngeal artery; T, thyroid cartilage.

**Figure 2:**
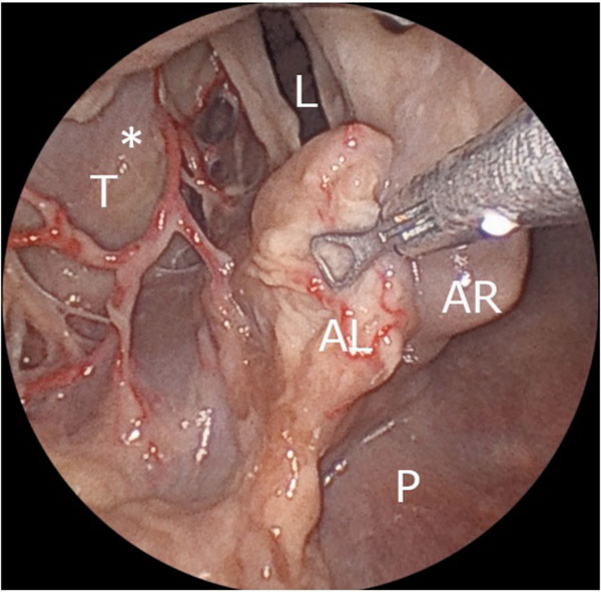
Left SLA separated from the perichondrium of the thyroid plate by a thin muscular layer (*). AL, left arytenoid area; AR, right arytenoid area; L, laryngeal lumen; P, pharyngeal lumen; SLA, superior laryngeal artery; T, thyroid cartilage.

After its entry into the larynx, the main trunk of the SLA ran ventro‐caudally while being tightly attached to the outer wall of the larynx. The course of the main trunk smoothly ran from cranial to caudal and we did not notice a “point of inflexion” in any of the 12 hemilarynges except in 1. In the other 11 specimens, the main trunk of the SLA rather descended obliquely (Figure 1). In all specimens, the main trunk of the SLA was separated from the inner perichondrium of the thyroid plate by a thin muscle layer derived from the inferior constrictor muscle of the pharynx (Figure 2).

After running in the paraglottic space for a variable distance, the SLA started to branch. It bifurcated at the supraglottic level into an ascending branch directed towards the epiglottis and showing a marked undulation (meandering) and a descending branch directed towards the laryngeal mucosa and the intrinsic muscles of the larynx. The descending branch bifurcated again into a terminal anterior and a terminal posterior branch. The level of this second bifurcation was variable. The terminal anterior branch coursed ventro‐caudally towards the anterior commissure and left the larynx through the cricothyroid membrane (Figure 2).

**Figure 3:**
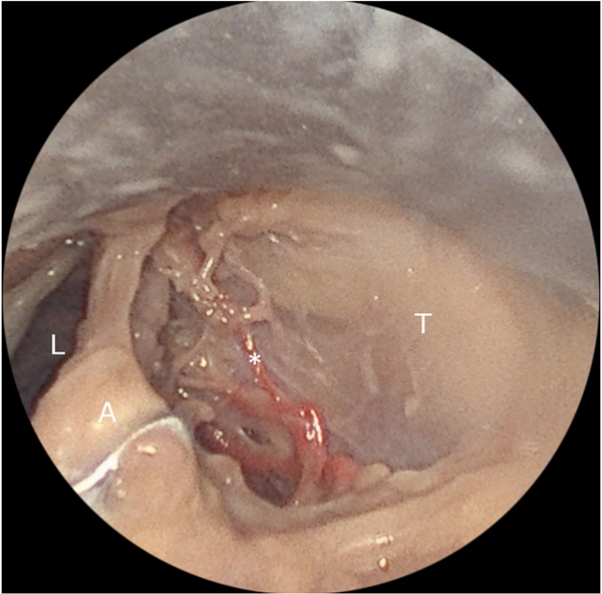
The epiglottis supplied by an ascending branch (*) from the anterior terminal division of the descending branch of the right SLA. A, right arytenoid area; L, Laryngeal lumen; SLA, superior laryngeal artery; T, thyroid cartilage.

**Figure 4:**
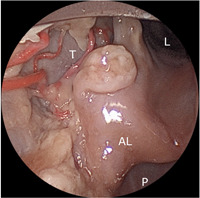
The arytenoid branch of the left SLA can be doubled (1, 2). AL, left arytenoid area; L, laryngeal lumen; P, pharyngeal lumen; SLA, superior laryngeal artery; T, thyroid cartilage.

**Figure 5:**
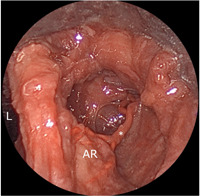
The arytenoid branch of the right SLA was ascending (*) in 1 cadaver. AR, right arytenoid area; L, laryngeal lumen, SLA, superior laryngeal artery.

The epiglottis was occasionally supplied by extra ascending branch(es) from the anterior division of the descending branch of the SLA (Figure 3). A branch of the SLA supplied the arytenoid area and showed considerable meandering. This branch was occasionally doubled (Figure 4) and had usually a descending course except in 1 cadaver where it ran an ascending course on both sides (Figure 5). Rusu et al reported that the branch of the SLA directed to the arytenoid is descending in 77% of his specimens and is ascending in 18.5%.^2^

**Figure 6:**
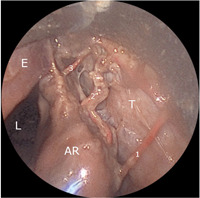
Three medial branches bridge the right paraglottic space from lateral to medial (1, 2, 3). AR, right arytenoid area; E, epiglottis; L, laryngeal lumen; T, thyroid cartilage.

**Figure 7:**
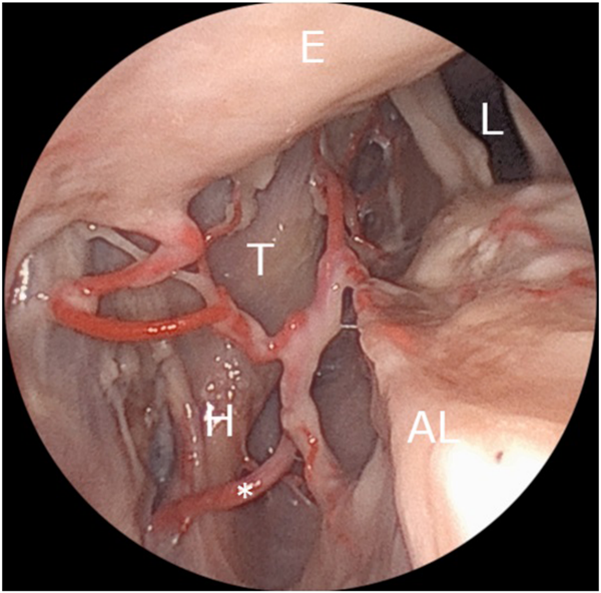
A branch from the left SLA runs caudally (*) to leave the larynx laterally around the lower horn (H) of the thyroid cartilage. AL, left arytenoid area; E, epiglottis; L, laryngeal lumen; SLA, superior laryngeal artery; T, thyroid cartilage.

One or more medial branches of the SLA bridged the paraglottic space from lateral to medial on their ways towards the ventricle, the false and true vocal folds (Figure 6). A branch from the main trunk of the SLA ran caudally and laterally and left the larynx in close proximity to the lower horn of the thyroid cartilage (Figure 7).

Upon reviewing the anatomic description of the branches of the SLA elaborated by various authors, we deduced that the branch Rusu et al,^2^ designated as the superior branch, nearly coincides with the ascending branch according to Oki, Pearson, Sato, and Imanishi et al^1^, ^4^, ^5^, ^6^ and with the superior posterior branch described by Imura et al.^7^ Similarly, the anterior branch described by Rusu et al^2^ and by Imura et al^7^ corresponds to the ventral branch according to Oki, Pearson, and Sato.^4^, ^5^, ^6^ The posteromedial branch described by Rusu et al,^2^ corresponds to the medial posterior branch described by Imura^7^ and to the dorsal branch according to Oki, Pearson, and Sato.^4^, ^5^, ^6^ Table 1 correlates the names of the constant branches of the SLA cited in previous publications to their areas of distribution.

**Table 1:** Correlation between the names of the main branches of the SLA and their areas of distribution according to authors of previous publications.

Area of distribution	Branch name according to authors of previous articles						
	Oki, Pearson, and Sato^4^, ^5^, ^6^		Imanishi^1^		Imura^7^	Rusu^2^	Perotti^8^
Epiglottis	Ascending		Ascending		Superior posterior	Superior	Epiglottic artery
Ventricle	Ventral				Anterior	Anterior	
Posterior wall of larynx and anterior wall of laryngo‐pharynx	Dorsal				Medial posterior	Postero‐medial	
Ventricular fold	Medial			Descending	Medial		
Thyroarytenoid muscle (vocal fold) and more caudal		Descending Anterior division		Anterior division	Antero‐inferior	Antero‐inferior	Antero‐inferior artery
		Posterior division		Posterior division	Postero‐inferior	Postero‐inferior	Postero‐ inferior artery

Abbreviation: SLA, superior laryngeal artery.

## Discussion

Postoperative hemorrhage from the SLA or its branches is a major complication after open laryngeal surgery. It is specially more serious when it occurs after TLM or TORS because the bleeding takes place in unprotected airways, not secured by a tracheostomy.^6^, ^7^ Such a complication is difficult to predict and its incidence ranges from 0.6% to 8% after TLM.^8^ It may reach up to 14% if supraglottic laryngectomy is included.^3^

We did our inside out dissection of the SLA on specimens that were fresh frozen and not formalin fixed. Compared to fixed ones, fresh frozen specimens are closer to depict the normal human anatomy.^8^ In order to preserve the natural fixation point of the vessels,^10^ we did not dissect or remove the thyroid cartilages from their genuine anatomic positions and we did not extract the larynges from the necks.

While describing the intralaryngeal course of the SLA, Rusu et al^2^ advocated that the point of inflexion from the transverse course of the SLA to its descending vertical one is located anterior to the base of the superior horn of the thyroid cartilage. From the inside aspect, we did not notice such an inflexion point except in only 1 out of the 12 hemilarynges. The interpretation of this discrepancy between our findings and those from the above‐mentioned authors may be that 8 of our 12 hemilarynges were from females, where the thyroid laminae are too short^6^ to allow for such an inflexion.

Souviron et al,^11^ microdissected 20 formalin‐fixed cadaveric larynges through an endoluminal approach. They claimed that the superior laryngeal vessels were located under the mucosa of the superior third of a triangle limited by the epiglottic attachment with the aryepiglottic fold, the anterior commissure, and the apex of the vocal process. They proposed that this triangle is the landmark for identification and clamping of the neurovascular elements in the supraglottis. Based upon our dissection and upon the data extracted from Goyal et al^3^ and Sato,^6^ we do not agree that the artery designated by Souviron et al, is the main trunk of the SLA. It is rather its superior (ascending or epiglottic) branch. At the said level, the SLA main trunk is located at a more dorsal plan,^6^ being tightly attached to the thyroid cartilage inner lamina laterally.

In the literature, the anatomic description of the SLA branches showed wide nomenclature discrepancies^2^, ^3^, ^7^, ^8^, ^9^, ^10^, ^11^ secondary to a lack of consensus on its branches' naming. We agree with Rusu et al that “the branching pattern of the SLA must be re‐discussed.”^2^ We agree also that an exhaustive description of all possible branches of the SLA is of minimal surgical interest. Goyal et al focused only on the main trunk of the SLA beside the SLA branches distributed to the epiglottis and arytenoids.^3^ Perotti et al highlighted only 3 branches of the SLA, namely: the epiglottic, the antero‐inferior, and postero‐inferior arteries. Other than that, they described an anastomotic network in the paraglottic space between the antero‐inferior and the postero‐inferior arteries.^8^

Pearson stated, decades ago, that the branches of the SLA arose in many orders and combinations, but were distributed to fairly constant locations.^5^ Accordingly, and based upon the comparison we did between the different names of the SLA branches (Table 1), we suggest to name the main branches of the SLA according to their distribution, that is, their area of supply, instead of nominating them according to their directions. A more easily understandable designation would be “the epiglottic branch,” “the arytenoid branch,” “the muscular branches,” “the luminal mucosal branches,” and so forth. Pearson already called the ascending branch “the epiglottic branch.”^5^ He also stated that many “unnamed arterial branches and arterioles“ are present.^5^

## Conclusion

The inside out surgical anatomy of the SLA and of its main branches deserves a pertinent knowledge to prevent and manage intraoperative and postoperative bleeding after TLM or TORS. An exhaustive anatomic description of all the branches of the SLA is, however, of minimal surgical importance. Naming of the main branches of the SLA according to their distribution would resolve much of the ambiguities surrounding the SLA branches' nomenclatures.

## Author Contributions

**Hazem M.A. Saleh**, design of the work, data acquisition and analysis, drafting and revision of the article and figures, submission of the final version; **Thomas Jöns**, design of the work, revision of the article, approval of the final version; **Dirk Mürbe**, design of the work, revision of the article, approval of the final version; **Tadeus Nawka**, design of the work, data analysis, drafting the figures, revision of the article, approval of the final version.

## Disclosures

### Competing interests

None.

### Funding

Professor Hazem M.A. Saleh received a short‐term scholarship from The German Academic Exchange Service (DAAD)/German Egyptian Research Short‐Term Scholarship Programme (GERSS), 2020 Grant # (57522433).
